# Relationship between maternal and infant serum vitamin D levels in Jos, Plateau State, Nigeria: a cross-sectional study

**DOI:** 10.11604/pamj.2023.46.48.37578

**Published:** 2023-10-02

**Authors:** Ishaya Ibrahim Abok, Lucius Chidiebere Imoh, Fidelia Bode-Thomas, Stephen Oguche, Ayuba Zoakah, Atiene Sagay

**Affiliations:** 1 Department of Paediatrics, College of Health Sciences, University of Jos, Jos, Nigeria,; 2 Chemical Pathology, College of Health Sciences, University of Jos, Jos, Nigeria,; 3 Department of Community Medicine, College of Health Sciences, University of Jos, Jos, Nigeria,; 4 Department of Obstetrics and Gynaecology, College of Health Sciences, University of Jos, Jos, Nigeria

**Keywords:** Mother, infant, 25 hydroxyl vitamin D_3_, Nigeria, vitamin D

## Abstract

**Introduction:**

there is growing interest in the link between maternal and infant vitamin D (VD) levels. Breast milk transmission and the fact that the mother and her child may share risk factors for VD, such as exposure to sunlight, diet, and sociocultural influences may impact VD status, the magnitude of which is largely unknown in our topical low-middle income setting. We assessed the connection between maternal and infant VD status including their correlates.

**Methods:**

this cross-sectional study investigated 95 maternal-infant pairs in Jos. Mothers were interviewed using a questionnaire. Blood sampling and analysis of serum total 25 hydroxy VD were performed using the chemiluminescent immunoassay method. Maternal and infant VD levels were classified as VD deficient (VDD), VD insufficient (VDI), and VD sufficient (VDS). The mean maternal and infant VD were compared, and the Spearman correlation between them was assessed, a stepwise linear regression was also performed with infant vitamin D as a dependent variable. For all statistical analysis, p<0.05 was considered significant.

**Results:**

the median maternal and infant VD was 29.68 ng/ml and 29.41 ng/ml, respectively. The mean infant VD (32.19 ± 10.61 ng/ml) was comparable to maternal VD (31.12 ± 12.94 ng/ml) (p=0.483), with a Spearman correlation coefficient of 0.3 (p=0.037). Maternal vitamin D (beta=0.539, duration of exclusive breastfeeding (beta=-3.490), and infant age (beta=1.655) were found to be significant independent predictors of infant vitamin.

**Conclusion:**

beyond neonatal age, a significant positive relationship between maternal and infants´ VD levels exists and suggests that family-focused vitamin D intervention might be an effective public health approach in the tropical city of Jos.

## Introduction

The role of vitamin D (VD) in maintaining good health is well established in humans; these include bone mineralization, promotion of perinatal growth, modulation of the defense system against some non-communicable (such as diabetes), and some infectious diseases such as acute respiratory tract infections (ARI) [1-7]. Humans acquire ninety percent of their daily requirement of VD through photo-biosynthesis following exposure of their skin to ultraviolet light B (UVB) sunlight. Dietary intake of VD accounts for the remaining ten percent. In fetuses, the transplacental transfer of VD is the only source, and postnatally, transfer through the breast milk remains vital. Because of this maternal-infant relationship, optimizing VD through maternal or newborn supplementation is a strategic practice in some developed temperate countries [8-14].

Studies suggest some correlation between maternal VD and newborn VD [15-18]. In addition, similarities in mother-newborn pair mean VD levels, and the proportions of VD sufficient and deficient subjects have previously been established [15-18]. However, it is unclear if this relationship extends beyond the neonatal period, into later infancy (1-12 months). This question is vital because the maternal transfer of VD in infants continues via breast milk, even though minimal and the mother-infant pair are bound to share similar physical and social environmental determinants of VD such as access to sunlight and the consumption of VD-rich foods [19]. However, studies on infant VD status and its correlation with their lactating maternal vitamin D are lacking in the sub-Saharan African tropical environment. Therefore, this study sought to determine the relationship between infants and their mothers´ serum total 25 hydroxy VD levels. Specifically, we try to examine the correlation between maternal and infant serum vitamin D levels, the factors associated with low vitamin D levels in the mothers and their infants as well as maternal and infant factors that predict infant vitamin D. This information will offer insight into the health promotive approach that can reduce the negative influences of VDD among children in Jos.

## Methods

**Study site/area:** the study site, the Jos University Teaching Hospital (JUTH), is located in Jos, the capital city of Plateau State, North-Central, Nigeria. Jos is a highland area at 1,217 meters above sea level. The climate is tropical savannah, and the area receives 2,672 hours of sunlight per year with temperatures between 11°C and 23°C; its coordinates are latitude 9° 56´ N and longitude 8° 53´ E [20].

The study participants were mothers and their infants attending the Family Health Clinic and Immunization Clinic at JUTH. The clinic is managed by the Community and Public Health Department of JUTH. Mothers who present in the clinic with their under-five children are first registered, then their weight, length, and temperature are assessed and documented in the child's health record by a health worker. Afterward, all mothers are given a health talk in the waiting hall by a community health officer or a community health physician. Thereafter, each of the mothers is directed to one of three consulting rooms to be seen by a public health physician. The final stage is the receipt of immunizations for infants who require them.

**Study design and study population:** this is a single-facility cross-sectional study. All consenting biological mothers who were the primary caregivers of their infants and all clinically health infants aged 1-12 were included in the study. Non-biological mothers, and children brought up primarily by nannies or relatives were excluded. Also excluded were infants that had clinical evidence of chronic diseases such as liver and kidney diseases, extensive dermatologic diseases, and malformations unknown birthweight.

**Sample size:** the sample size was determined using online sample size calculator for comparing means between two independent groups [21]. Using a mean VD in non-pregnant women 23.7 ± 1.4 ng/ml and healthy under five 26.0 ± 8.6 ng/ml [22,23]. After inputting a mean difference of 2.3 as effect size (mean in group 1 minus mean in group 2) and inputting a standard deviation of 5.5ng/dl (pooled standard deviation of group 1 and group 2), a power of 0.8, 0.05 as precision, a sample size of 90 per group was obtained.

**Sampling technique:** on clinic days, the researcher and assistant address all mothers and infants after the group health talk in the waiting room. The aim was to identify all infants aged 1 month to 12 months. All identified mothers were given a ballot paper, and any mother who chose a paper with a serial number written on it was enrolled in the study if both the mother and infant met the inclusion criteria.

**Procedure for data collection:** after enrollment, data was obtained from the mothers using a structured questionnaire administered by the researcher. Clinical examination and parameters were measured and documented. Thereafter, 2 mls of blood was collected from both mother and baby. Data collection tool: a structured questionnaire was used for data collection. The questionnaire has a section on maternal variables and a section on infants. Maternal information that was collected using the questionnaire included sociodemographic variables such as maternal education, place of residence, and type of housing, as well as pregnancy-related information such as duration of hospitalization during pregnancy, parity, drugs used in pregnancy, use of the hijab (a head covering worn in public by some Muslim women), and consumption of milk, eggs, and oily fish such as salmon, tuna, and sardines. The maternal weight, height, and body mass index (BMI) were assessed. Underweight mothers had a BMI of less than 20 kg/m^2^, and normal BMI was 20-24.99 kg/m^2^, overweight was 25-29.9 kg/m^2^, and severe obesity was 30 kg/m^2^.

The infant information assessed using the questionnaire included birthweight, hospitalization, and phototherapy during the neonatal period, during which the infant was kept completely indoors without exposure to the sun, the number of hours spent outdoors per day, the type of housing, and the feeding practices, i.e. the duration of exclusive breastfeeding (EBF). The infant's exposed body surface area (BSA) was measured using the Lund nomogram. Anthropometric measures of weight (kg) and length (cm).

**Blood sampling:** using standard procedure, blood samples were taken from each mother and her infant and collected into a plain sample, and centrifuged at 4000 rpm for 5 minutes to obtain serum for analysis. The serum was collected into cryovials and stored at -20°C until analysis at three months. Serum total 25-hydroxy-VD was carried out using the electrochemiluminescence binding assay method on an automated COBAS E411 immunochemistry analyzer (Roche Diagnostics, Mannheim, Germany) [24]. The analysis was done at the Department of Chemical Pathology Laboratory of JUTH.

**Ethical consideration:** written informed consent was obtained from each mother before enrollment of the mother-baby pair into the study. The Ethics and Research Committee of the Jos University Teaching Hospital approved the study with the ethical reference number: JUTH/DCS/ADM/127/XXVII/835.

**Statistical analysis:** the participant's variables were summarized as proportions, median and interquartile range, mean and standard deviation, frequency, and percentages. Vitamin D was categorized as vitamin D deficiency (VDD) if <20 ng/ml, vitamin D insufficiency (VDI) if between 20 to <30 ng/ml and vitamin D sufficiency (VDS) if ≥30 ng/ml, and at risk of toxicity if >150 ng/ml [2]. The participants were further categorized into two groups: those with low vitamin D (vitamin D insufficiency and deficiency) and those with normal vitamin D (VDS).

The student t-test, tested for the difference in maternal mean VD and infant mean vitamin D, and the Spearman test assessed for the correlation between infant and maternal vitamin D. To determine if infants' vitamin D is influenced by maternal vitamin D, a multivariable stepwise linear regression was done, and the final model was reported. In the linear regression model, infant vitamin D level was the outcome, while maternal continuous variables such as maternal vitamin D, maternal BMI, and duration of hospitalization during pregnancy were the dependent variables. Other variables included birth weight, duration of hospitalization during the neonatal period, current weight, length, head circumference, size of the anterior fontanel, number of erupted teeth, duration of exclusive breastfeeding, and proportion of body surface that was unclothed. All descriptive results were presented as prose, tables, and figures. The criterion for significance for all analyses was set at a P-value < 0.05. All statistical analyses were carried out using SPSS version 25.

**Funding:** this work was supported by the Support of Training and Mentoring in Nigeria for Academics (STAMINA) D43TW010130 grant.

## Results

A total of 95 mothers and their infants were enrolled.

**Maternal characteristic:** the parity of the mothers who took part was as follows: 31 (32.6%) were primiparous, 19 (20.0%) had parity 2, 16 (16.8%) had parity 3, 7 (7.4%) had parity 4, and the remaining 22 (23.2%) had multiparity. Hijab was frequently used by 32 (33.7%) of the mothers; the majority (43.2% of the mothers) had a secondary level of education and 26.3% had a tertiary level of education. Sixteen (16.8%) lived in rural areas, while only six (6.3%%) live in multistory buildings.

**Figure 1:**
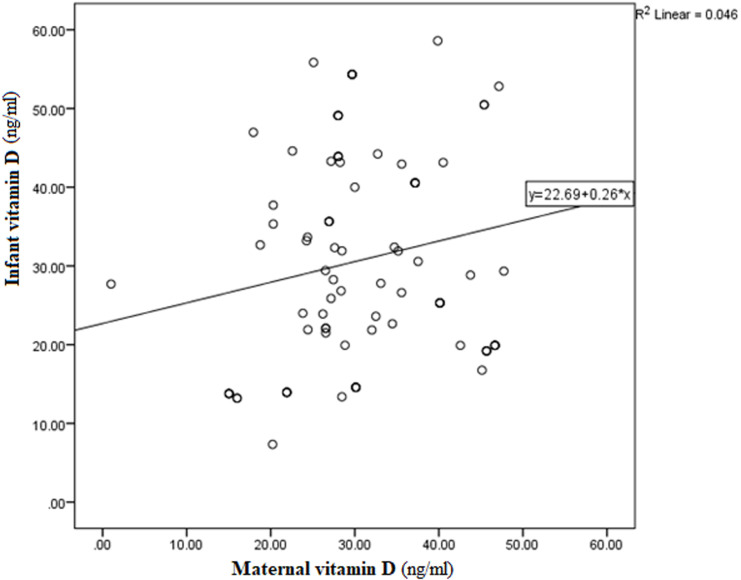
scatter plot showing the partial regression plot between maternal and infant vitamin D concentration

**Maternal vitamin D:** the median maternal serum vitamin D level was 29.68 ng/ml, with the 25^th^ and 75^th^ percentiles being 26.2 and 40.12 ng/ml, respectively, giving an interquartile range of 13.92 ng/ml. With respect to VD status, as shown in Figure 1, 46.8% (46) of mothers were sufficient, 42.1% (40) were insufficient, and 9.5% (9) were deficient. Regarding factors associated with low VD in the mothers, as shown in Table 1 mothers who consumed on average 4.14 eggs per week had normal VD compared to mothers who ate less, 2.27; p=0.0001. The odds of low VD in Mothers who ate eggs ≤2 days a week was 5.24.

**Table 1:** the association of some infant’s characteristics and infant vitamin D status

Maternal characteristics		Low VD n=48 (%)	Normal VD n=47 (%)	p-value	OR (95% CI)
Christian religion	No	31 (56.4)	24 (43.6)	0.274	1.58 (0.70 - 3.58)
	Yes	18 (45.0)	22 (55.0)		
Density of locality	Low	20 (55.6)	16 (44.4)	0.545	1.29 (0.56 - 2.97)
	High	29 (49.2)	30 (50.8)		
Urban	No	11 (64.7)	6 (35.3)	0.232	1.93 (0.65 - 5.74)
	Yes	38 (48.7)	40 (51.3)		
Hijab	No	28 (44.4)	35 (55.6)	0.051	0.42 (0.17 - 1.01)
	Yes	21 (65.6)	11 (48.4)		
Employment	No	16 (61.5)	10 (38.5)	0.233	1.75 (0.70 - 4.38)
	Yes	33 (47.8)	36 (52.2)		
Normal weight	No	24 (45.3)	29 (54.7)	0.186	0.56 (0.24 - 1.32)
	Yes	22 (59.5)	15 (40.5)		
Multi-vitamin	No	34 (46.6)	39 (53.4)	0.075	0.41 (0.15 - 1.12)
	Yes	15 (68.2)	7 (31.8)		
Storey building	No	44 (49.4)	45 (50.6)	0.236	0.20 (0.02 - 1.74)
	Yes	5 (83.3)	1 (16.7)		
Tertiary education	No	36 (52.2)	33 (47.8)	0.850	1.09 (0.44 - 2.69)
	Yes	13 (50.0)	13 (50.0)		
Eggs (days/week)	≤ 2	33 (71.7)	13 (28.3)	<0.001	5.24 (2.18 - 12.58)
	> 2	16 (32.7)	33 (67.3)		
Oily fish consumption	No	21 (60.0)	14 (40.0)	0.210	1.71 (0.74 - 3.99)
	Yes	28 (46.7)	32 (53.3)		
Family size	≤ 6	22 (52.4)	20 (47.6)	0.889	1.06 (0.47 - 2.38)
	> 6	27 (50.9)	26 (49.1)		

OR: odd ratio; VD: vitamin D

**Infants´ characteristics:** the infants enrolled were aged 1-12 months. The median age was 2.5 months, and the interquartile range was between 2 and 4.4 months for the infants. There was no statistical difference between the mean age in months of males (3.5 ± 2.7) and females (4.2 ± 2.9) (p=0.2244). Of the 95 infants, 32 (33.4%) were female, and the rest were male. Of the 95 children, 22.1% (22) were delivered through caesarean section; 69.5% (66) were normal birth weight at birth, while 8 children had no birthweight on their health record and the rest were low birth weight. Only 14.7 (14) infants received phototherapy during the neonatal period, and 28.4% (27) had been hospitalized previously, while 23.2% of children were on multivitamins.

**Figure 2:**
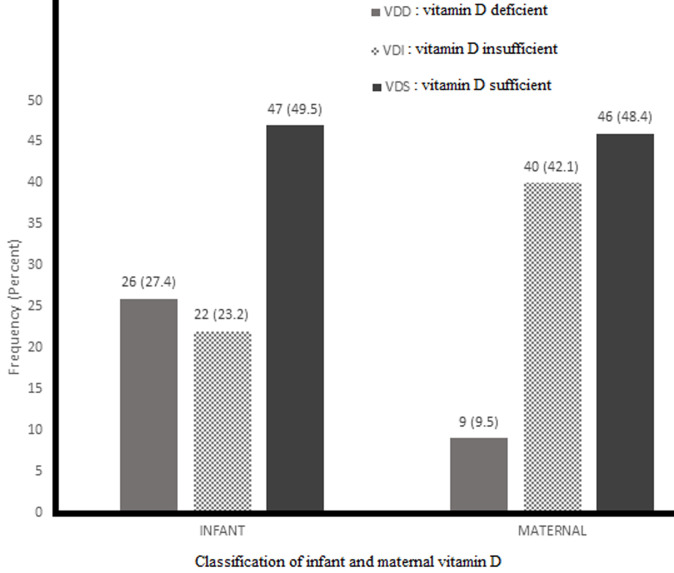
a bar chart of showing the frequency of vitamin D deficient (VDD), vitamin D insufficient (VDI), vitamin D sufficient (VDS) of infant and mother

**Infants vitamin D:** Figure 2 shows that out of the ninety-five infants, 47 (49.47%) were VDS, 22 (23.16%) were VDI, and 26 (27.36%) were VDD. The median infant serum vitamin D concentration was 29.41 ng/ml, and the 25^th^ and 75^th^ percentiles were 19.90 and 40.55 ng/ml, respectively, giving an interquartile range of 20.65 ng/ml.

As shown in Table 2, the odds of low VD in female infants was 7.306. The odd of low VD was 0.154 and 0.500 if an infant was delivered through caesarean section and was low birth weight at birth respectively. There was no difference in mean age of infants with low VD was 4 ± 2.8 months compared to 3.4 ± 2.8 months in infants with normal VD (p=0.294). There was also no difference (p=0.435) between the body surface area (BSA) that was not covered with clothing when the child was enrolled of infants with low VD (14.3 ± 6.6 M^2^) and infants with normal VD (15.5 ± 8.0 M^2^). There was also no difference (p=0.385) between the number of weeks the child was kept indoors following delivery of infants with low VD (5.1 ± 3.0 weeks) and infants with normal VD (5.6 ± 2.4 weeks).

**Table 2:** the association of some maternal characteristic and maternal vitamin D status

Infant characteristics		Low VD n=48 (%)	Normal VD n=47 (%)	p-value	OR (95% CI)
Gender	Female	10 (33.3)	20 (66.7)	0.023	0.36(0.14 - 0.88)
	Male	38 (58.5)	27 (41.5)		
CS delivery	No	35 (46.7)	40 (53.3)	0.145	0.47 0.17 - 1.31)
	Yes	13 (65.0)	7 (35.0)		
Low Birthweight	No	37 (50.0)	37 (50.0)	0.847	0.91 (0.35 - 2.40)
	Yes	11 (52.4)	10 (47.6)		
Underweight	No	32 (43.2)	42 (56.8)	0.008	0.16 (0.04 - 0.62)
	Yes	14 (82.4)	3 (17.6)		
Normal weight	No	15 (75.0)	5 (25.0)	0.027	3.82 (1.26 - 11.59)
	Yes	33 (44.0)	42 (56.0)		
Hospitalization	No	32 (47.1)	36 (52.9)	0.283	0.61 (0.25 - 1.51)
	Yes	16 (59.3)	11 (40.7)		
Phototherapy	No	41 (50.6)	40 (49.4)	0.966	1.03 (0.33 - 3.19)
	Yes	7 (50.0)	7 (50.0)		
Breast feeding type	EBF	42 (50.0)	42 (50.0)	1.000	0.83 (0.24 - 2.94)
	PEBF	6 (54.5)	5 (45.5)		
BMS	No	42 (53.8)	36 (46.2)	0.166	2.14 (0.72 - 6.36)
	Yes	6 (35.3)	11 (64.7)		
Multi-vitamin	No	44 (60.3)	29 (39.7)	0.001	6.83 (2.10 - 22.23)
	Yes	4 (18.2)	18 (81.8)		
Density of locality	Low	13 (36.1)	23 (63.9)	0.028	0.39 (0.17 - 0.91)
	High	35 (59.3)	24 (40.7)		
Urban	No	13 (76.5)	4 (23.5)	0.036	3.99 (1.20 - 13.34)
	Yes	35 (44.9)	43 (55.1)		

OR: Odd Ratio; VD: Vitamin D; CS: caesarean section; BMS: breast milk subtitute; EBF: exclusive breastfeeding; PEBF: predominant exclusive breast feeding;

**Comparison of mean infant and maternal vitamin D:** the infant's mean vitamin D level was 32.19 ± 10.61 ng/ml, compared to a mean maternal VD of 31.12 ± 12.94 ng/ml. The mean difference of 1.07 was not statistically significant (p=0.483).

**Correlation between maternal and infant vitamin D:** the Spearman correlation coefficient of 0.27 was statistically significant at p=0.037 (Figure 1).

**Multivariate analysis:** the multivariate stepwise linear regression model showed that the model was statistically significant (F=12.472; p<0.0001). Maternal vitamin D, infant age, and duration of exclusive breastfeeding were all independent predictors of the infant's vitamin D status (Table 3). These three predictors account for 40.1% of the variation in infant vitamin D levels, R squared=0.41. The model showed that a one unit increase in maternal vitamin D increased infants' vitamin D by 0.539, a one-unit increase in the duration of exclusive breastfeeding increased infant vitamin D by -3.490, and a one-unit increase in age increased infant vitamin D by 1.655. The following factors: birth weight (p=0.664), weeks spent outdoors (p=0.817), weight (p=0.362), length of the infant (0.788), size of the anterior fontanelles (0.788), exposed body surface area (0.895), and maternal weight (0.658) were not significant predictors of vitamin D in infants. Figure 1 shows that maternal vitamin D in isolation accounts for just 6% of the variation in Infant vitamin D in a linear plot.

**Table 3:** linear multivariate analysis of predictors of infant vitamin D

Model	Unstandardized coefficients		Standardized coefficients	t	Sig.	95.0% confidence interval for B	
	B	Std. error	Beta			Lower bound	Upper bound
Constant	15.185	7.929		1.915	0.059	-0.572	30.943
MvitD	0.187	0.110	0.154	1.693	0.094	-0.032	0.406
Age	-0.763	0.689	-0.163	-1.107	0.271	-2.133	0.606
Gender	-7.586	2.555	-0.272	-2.969	0.004	-12.665	-2.508
MVT	14.575	2.938	0.479	4.962	0.000	8.737	20.413
Weight	2.532	1.478	0.259	1.713	0.090	-0.406	5.469

**OR**: odd ratio; **VD**: vitamin D, **MVT:** multivitamin; **MVitD**: maternal vitamin D

## Discussion

This study showed that there is a link between the VD status of breast-feeding mothers and that of their infants. Also, it found that the relationship between VD and duration of exclusive breastfeeding is inverse but increases with increasing age. Furthermore, vitamin D deficiency (VDD) was found in nine percent of mothers, and this figure increases to fifty-one percent when the percentages of mothers with insufficient VD and mothers with VDD are added together. Infants and mothers exhibited similar proportions of individuals with VDD, hypovitaminosis D, VDS, and mean VD concentrations.

The mothers in this study were breastfeeding mothers; previous Nigerian studies had taken samples from mothers who had recently given birth [15,16]. Vitamin D deficiency was found in 9.1 percent of breastfeeding mothers in this study, compared to 4.7 percent and 20.2 percent of new mothers in Lagos and Maiduguri, respectively [15,16]. Although the comparison of nursing and just-delivery women is debatable, both groups of mothers are advised to consume the same amount of VD daily. In temperate nations, VDD is more common in breastfeeding mothers with a prevalence of 75.4 percent [25-27].

However, the participants in this current study had a larger proportion of mothers who are vitamin D sufficient (48.4 percent) compared to mothers in Europe (5.6 - 22 percent) [26,27]. This disparity is expected because Jos is a tropical location, that receives more hours of sunlight per year (2672 hours) than temperate countries like Germany (1896 hours) [20,28]. Because UV sunshine is required for VD production by the skin, enough sunlight is frequently one of the most important environmental elements that promote vitamin D sufficiency [1].

Despite this advantage in Jos Plateau State, low VD was seen in half of the mothers in this study. This shows that a barrier to sunlight availability could be a significant factor [29]. Although the causes for insufficient availability to sunshine are beyond the focus of this research, there are some explanations for the high degree of hypovitaminosis D found in our current study. According to the findings of this study, 75% of mothers with normal VD did not wear hijab, a type of excessive body covering that prevents direct synthesis of VD by the skin following UVB exposure on the skin. Maternal vitamin D deficiency was linked to extra body covering in a comparable study involving a population of mother and her child [15,16,29]. Excessive body covering hides the skin and reduces the amount of body surface area exposed to UV light, which increases photolysis of 7 hydro cholesterol in the epidermis and sets the stage for VD formation via several metabolic pathways [1]. Furthermore, in this study, just 1% of mothers received VD supplementation, compared to around 30% in Lagos and Sweden [15,27]. The combination of extensive body covering and inadequate VD supplementation could explain why only a small fraction of women experienced hypovitaminosis D in Lagos compared to the mothers in this study. More research in our sun-rich environment is needed to investigate this pattern and develop possible preventive approaches such as health education and VD information dissemination.

This study's infant mean VD (32.19 ± 10.61 ng/ml) is appropriate and comparable to that of other infants in sub-Saharan Africa [22,29]. This concentration is physiologically adequate for bone mineralization (not less than 10ng/dl) [1,2]. In South African Caucasian well-nourished 6 weeks infants, whose mothers had no VD supplementation, lower mean VD (1.1 ± 1.4 ng/ml) has been reported [30]. The research was conducted in South Africa during the winter, when it is cold, and as a result, individuals and their families have a tendency to overly cover themselves and stay indoors to avoid the cold. Although the average daily sunshine in South Africa during the study was 8.7 hours, comparable to the sunshine in Jos, the sunshine was probably not accessible to the participants. Although vitamin D photobiosynthesis is faster in Caucasian non-melanin skin compared to African melanin-containing skin, the higher risk of sunlight injury and cancer in non-melanin skin may deter parents from exposing their infants to direct sunlight which is standard of care [31]. Furthermore, the higher maternal VD demonstrated in this current study may further explain why infants in this current study had a higher mean VD compared to their counterparts in South Africa.

The positive relationship between lactating maternal VD and infant-paired vitamin D was demonstrated. In this study in three levels, first there was similarity in mean vitamin D, secondly, there was a positive correlation and thirdly, maternal vitamin D was an independent predictor of infant vitamin D in addition to other two variables one of which is EBF that is directly related to the mother. A 1 ng/ml increase in serum maternal vitamin D increases the infant´s serum vitamin D by 0.539 ng/dl. In a study in South Africa, a similar relationship was demonstrated, mothers with lower VD had infants with lower VD, mothers who were supplemented with either 500 IU or 1000 IU had higher VD and their infants also had higher VD [29]. Therefore, optimizing maternal VD is also an approach to optimizing infant VD although infant VD supplementation is also an option, but maternal supplementation is helpful in optimizing foetal VD in utero [7].

**Limitations:** this study provided important insight into VD of maternal-infant pairs; however, the study population was hospital-based, so it is possible specific mothers may have a preference for specific health care facilities. A community-based study will better represent the population.

## Conclusion

This study discovered a link between maternal and infant VD concentrations, as well as low vitamin D levels in roughly half of the mother-infant pairs, indicating that a similar proportion of mothers and infants were vitamin D sufficient. This means that vitamin D deficiency is a public health concern for African mothers and children living in tropical environments and that a maternal-infant approach to prevention may be effective. As a result, the data from this study can be used as background data for larger community-based studies.

### What is known about this topic

- *Previous studies in Nigeria used cord blood to assess newborn vitamin D and demonstrate a strong correlation between mother-newborn pairs;*

### What this study adds

- *The relationship between maternal and infant continues even after the neonatal period;*
- *The correlation becomes moderate during the infancy age 1-12 months and higher maternal VD infers higher infant VD;*
- *That about forty percent of the variance in maternal and infant vitamin D can be attributed to three variables of which two are maternal, maternal vitamin D, and duration of exclusive breastfeeding; insights into the vitamin D status of mothers and infants in Jos, Nigeria*.
